# Application of ^13^C Quantitative NMR Spectroscopy to Isotopic Analyses for Vanillin Authentication Source †

**DOI:** 10.3390/foods10112635

**Published:** 2021-10-30

**Authors:** Concetta Pironti, Maria Ricciardi, Oriana Motta, Federica Camin, Luana Bontempo, Antonio Proto

**Affiliations:** 1 Department of Medicine and Surgery, University of Salerno, via S. Allende, 84081 Baronissi, SA, Italy; cpironti@unisa.it (C.P.); mricciardi@unisa.it (M.R.); 2 Fondazione Edmund Mach, Research and Innovation Center, Food Quality and Nutrition Department, 38010 San Michele all’Adige, TN, Italy; federica.camin@fmach.it (F.C.); luana.bontempo@fmach.it (L.B.); 3 Centre Agriculture Food Environment C3A, University of Trento, 38010 San Michele all’Adige, TN, Italy; 4 International Atomic Energy Agency, IAEA, International Centre, P.O. Box 100, A-1400 Vienna, Austria; 5 Department of Chemistry and Biology, University of Salerno, via Giovanni Paolo II 132, 84084 Fisciano, SA, Italy; aproto@unisa.it

**Keywords:** carbon stable isotope ratio, food, IRMS, vanillin, NMR

## Abstract

The carbon stable isotope ratio (δ^13^C) is a valuable chemical parameter in the investigation of the geographic origin, quality, and authenticity of foods. The aim of this study is the evaluation of the feasibility of ^13^C-NMR (Nuclear Magnetic Resonance) spectroscopy to determine the carbon stable isotope ratio, at natural abundance, of small organic molecules, such as vanillin, without the use of IRMS (Isotope Ratio Mass Spectrometry). The determination of vanillin origin is an active task of research, and differentiating between its natural and artificial forms is important to guarantee the quality of food products. To reach our goal, nine vanillin samples were analyzed using both ^13^C quantitative NMR spectroscopy (under optimized experimental conditions) and IRMS, and the obtained δ^13^C values were compared using statistical analysis (linear regression, Bland–Altman plot, and ANOVA (analysis of variance)). The results of our study show that ^13^C-NMR spectroscopy can be used as a valuable alternative methodology to determine the bulk carbon isotope ratio and to identify the origin of vanillin. This makes it attractive for the analysis in the same experiment of site-specific and total isotope effects for testing authenticity, quality, and typicality of food samples. Moreover, the improvement of NMR spectroscopy makes it possible to avoid the influence of additives on carbon stable isotope ratio analysis and to clearly identify fraud and falsification in commercial samples.

## 1. Introduction

The carbon stable isotope ratio (expressed as δ^13^C) is considered a valuable chemical parameter for studying the geographic origin of food products as well as for checking food properties, such as typicality, quality, and authenticity, especially when conventional analytical methodologies provide ambiguous results [1,2,3,4,5]. Furthermore, its determination gives the most important information concerning falsifications, adulterations, and fraud in food products [6]. For example, this method is the official method applied by the IOV (International Organization of Vines and Wine) [7] to quantify the sugar added into wine [8,9]. Isotope ratio mass spectrometry (IRMS) is the official methodology for the analysis of carbon stable isotope ratio due to its high accuracy (0.1‰) and sensitivity (up to 0.01‰) [10]. However, it requires expensive equipment and skilled technicians and measures the overall δ^13^C of a sample, a value that can be affected by the presence of a small number of impurities in the analyte, thus sometimes making the results unreliable. In fact, a disadvantage of this technique is related to the matrix influence; in the presence of a complex matrix, a selective sample of preparation techniques in addition to an efficient separation system before IRMS analysis is required [11,12]. Due to the increasing interest in this field, different analytical methods have been implemented for isotopic analysis. For example, infrared-based spectroscopy, mid-infrared (MIR) laser spectroscopy, non-dispersive (NDIRS), and Fourier transform (FT-IR) have been successfully applied in this field with low cost and complexity [1,13,14,15,16,17,18]. The advantages of spectroscopy instrumentation are their very common presence in the laboratory, and they require a simple protocol to complete the analysis. Recently, and for the first time, we applied ^13^C-NMR spectroscopy to measure δ^13^C values of inorganic carbonates and bicarbonates [19] without the use of IRMS as a reference value of carbon stable isotope composition. The results obtained with this alternative method were not influenced by the purity of the sample because only signals related to the chemical of interest are considered for the calculation of δ^13^C. This aspect makes ^13^C-NMR-based methodologies very useful for investigating food falsifications and sophistication.

**Figure 1:**
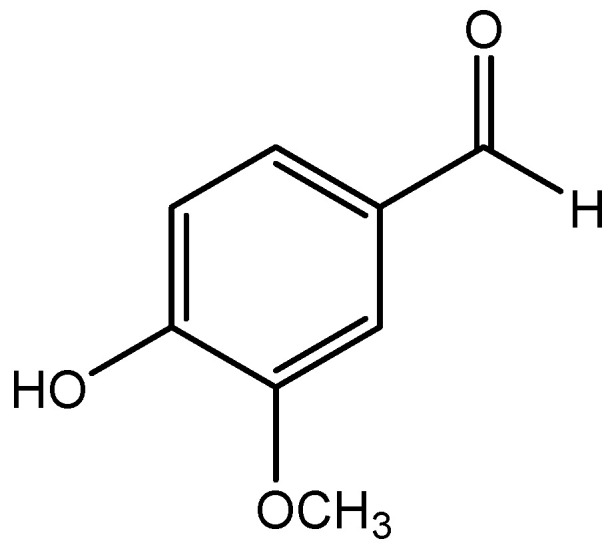
Chemical structure of 4-hydroxy-3-methoxybenzaldehyde, vanillin.

In recent years, carbon stable isotope analysis has been employed as a useful tool to differentiate between natural and artificial forms of vanilla extracts [20,21,22,23,24,25]. Vanilla is the second most expensive spice and is used as a flavoring component in the food industry. It is naturally obtained from cured vanilla beans coming from a tropical orchid plant [26]. Vanilla extracts are made of different polyphenols, which give them a pleasing aroma and flavor, together with more than 200 volatile compounds and a small number of tannins, waxes, and free amino acids. Vanillin (4-hydroxy-3-methoxybenzaldehyde, see Figure 1) is one of the main polyphenols in vanilla extract and the principal property responsible for its characteristic flavor and aroma. 

Natural vanillin is isolated from plants (i.e., vanilla beans) as a result of long and expensive processes, whereas artificial vanillin is produced using cheap precursors [27]. The vanillin is synthetized by an industrial process based on the condensation of guaiacol with glyoxylic acid, followed by an oxidation–decarboxylation step [28]. In addition, synthetic vanillin is also produced from lignin [29,30]. Firstly, an oxidant treatment of lignin in water solution is used (at high temperatures and pressures, and with alkaline pH) to depolymerize the lignin. Then, pure vanillin is isolated from the obtained complex mixture using different types of extractions [29]. However, the high demand for natural flavor induced an improvement in vanillin production from alternative natural sources, such as wheat, clove, turmeric, rice, corn, and sugar cane. Cheap syntheses of such bio-vanillin can be performed through a microbial and enzymatic transformation of natural raw materials, such as glucose [31], sucrose, ferulic acid, curcumin, eugenol, and isoeugenol [32]. 

**Figure 2:**
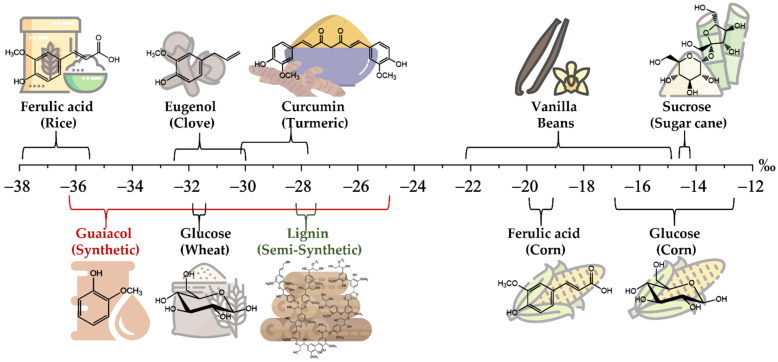
δ^13^C range of values for vanillin according to a specific source and chemical used as precursor.

In order to preserve and guarantee the quality of products, as well as to fight against mislabeling, which increases consumer distrust, it is fundamental to discriminate between artificial and natural forms of vanillin. In this context, the measure of the carbon stable isotope ratio (δ^13^C) becomes very useful. The δ^13^C value of a vanillin sample of natural origin is around −20‰, being a CAM (Crassulacean acid metabolism) plant extract, whereas values in the range from −36.2‰ to −24.9‰ are observed for samples of synthetic origins [23,33]. Considering all the possible routes for the synthesis of bio-vanillin, the δ^13^C range of values observed is from −37.8‰ to −12.5‰, depending on the specific source (rice, clove, wheat, turmeric, corn, and sugar cane) and the molecule used as a precursor (Figure 2) [34,35,36,37]. Moreover, the δ^13^C value of vanillin from vanilla beans ranges from −22.2‰ to −14.6‰ according to the type of plant (*Vanilla pompona*, *Vanilla planifolia,* and *Vanilla tahitensis*) and the geographic origin [23,34].

Although IRMS is the preferred method for the authentication of vanillin source, the δ^13^C measured can be easily manipulated by adding synthetic ^13^C-depleted vanillin or by mixing vanillin samples from different cheap sources. The IRMS method does not distinguish the carbon stable isotope ratio of the single compound from a complex matrix. In some cases, a purification pretreatment of samples with chromatographic methodologies is necessary [23,37]. Several scientific efforts are devoted to resolving this issue via the implementation of SNIF-NMR (site-specific natural isotopic fractionation by nuclear magnetic resonance) methodology [38,39]. Firstly, ^2^H, and recently ^13^C NMR, was shown to be more effective than the previous method, especially when an INEPT (insensitive nuclei enhancement by polarization transfer) sequence is used [20] due to the identification of a specific carbon stable isotope ratio of each signal in the spectra.

The aim of this study was the evaluation of the applicability of our recent employment of ^13^C-NMR spectroscopy in the discrimination of carbon isotopic composition of several commercially available vanillin samples. The use of this methodology could avoid the influence of the matrix on the determination of carbon stable isotope composition due to the use of an internal standard. Quantitative ^13^C-NMR spectroscopy (under optimized experimental conditions) was used to determine the bulk carbon stable isotope ratio of selected samples and to identify the origin of vanillin. Moreover, statistical analyses (linear regression, Bland–Altman plot, and ANOVA) were performed to compare NMR results with those obtained using IRMS and to validate our procedure.

## 2. Materials and Methods

### 2.1. Materials

This study was conducted on 9 selected samples of vanillin, of which 8 are commonly available on the market. To verify the reliability of the proposed method, a sample of natural vanillin (sample 9), i.e., extract from Vanilla plants, which has a known δ^13^C value (−20.3‰) [25], was analyzed. All samples were analyzed without any preliminary treatment. Chromium acetylacetonate (Cr(acac)_3_), acetone-d6 (>99.8% deuterated), and sodium acetate (CH_3_^13^CO_2_Na) were purchased from Sigma−Aldrich (Sigma Chemical Corp 3050 Spruce Street Saint Louis, MO 63103 United States St. Louis, MO, USA).

### 2.2. Carbon Stable Isotopic Analysis of Vanilla Samples by Elemental Analysis/Isotope Ratio Mass Spectrometry (EA/IRMS)

δ^13^C was measured using a Delta Plus V Isotope Ratio Mass Spectrometer (Thermo Fischer Scientific, Hanna-Kunath-Str. 11 28199, Bremen, Germany) with a Flash EA 1112 Elemental Analyzer (Thermo Fischer Scientific, Hanna-Kunath-Str. 11 28199, Bremen, Germany) [40]. To calculate the δ^13^C value, 2 homogenized in-house protein standards were used. The proteins were calibrated against international reference materials such as L-glutamic acid USGS 40 (United States Geological Survey—IAEA International Atomic Energy Agency, Vienna, Austria, δ^13^C = −26.39‰), fuel oil NBS-22 (National Bureau of Standards—IAEA, δ^13^C = −30.03‰), and sugar (IAEA-CH-6, δ^13^C = −10.45‰). The NORDTEST21-22 measures the internal reproducibility, and the uncertainty calculated for our analyses was 0.3‰. The international standard considered for δ^13^C values is the Vienna Pee Dee Belemnite, normalized according to a value of −46.6‰ to LSVEC lithium carbonate (IAEA) and +1.95‰ to NBS 19 calcium carbonate [41].

### 2.3. Characterization of Vanillin Samples with ^13^C-NMR Spectroscopy Analysis

The spectroscopy characterization was obtained using a Bruker 400 spectrometer, with tubes with an outer diameter of 10 mm. Quantitative NMR spectra were recorded using 0.1000 g of sample, 0.0100 g of internal standard (CH_3_^13^COONa), and 0.0050 g of relaxation reagent (Cr(acac)_3_), with the addition of 0.5 mL of acetone-d6. Quantitative analyses were performed by applying gated decoupling techniques and a pulse angle of 90° and pulse intervals (D) selected (D > 3T1, max) according to the longitudinal relaxation times (T1) (determined by the inversion recovery method). Experimental parameters were: spectrum width (SW) of 3000 MHz; 12 μs for pulse width; memory size (SI) of 32K; delay time, 150 s; 300 K, experimental temperature; zero filling (Z), 32K; 70 number of transients (NS); 3 experiments per sample (NE); time of analysis, 2 h. 

### 2.4. Expression of the Results

The carbon stable isotope ratio (expressed in δ relative to the reference material) is calculated using the following equation:

δ = (R_sample_ − R_reference_)/R_reference_ (1)

where the R value is an expression of ^13^C/^12^C. The δ values are multiplied by 1000 and expressed in units “per mil” ‰.

### 2.5. Statistical Analysis

The analysis of summary statistics, such as mean, standard deviation (SD), coefficient of variation, linear regression, and Bland–Altman plot, was performed using OriginPro software (2018 version). The correlation between IRMS and ^13^C-NMR was determined using linear regression analysis. A Bland–Altman plot was constructed by plotting the difference between the results of the two methods against their mean to evaluate the agreement between IRMS and ^13^C-NMR. Moreover, to assess the statistical differences between IRMS and ^13^C-NMR, one-way ANOVA was performed using R studio software (version 4.1.1). The null hypothesis for the ANOVA analysis was that the two methods show no differences in δ^13^C values for the same sample, so they can be used indiscriminately. In addition, one-way ANOVA was also performed for the origin of samples, using as the null hypothesis that the synthetic and natural samples show no differences in δ^13^C values. Therefore, the independent variables investigated were the “method of measure” (IRMS and ^13^C-NMR) and the “origin of vanillin” (synthetic and natural), while the dependent variable was the value of δ^13^C obtained from the measures. The significance level was α = 0.01.

## 3. Results 

**Figure 3:**
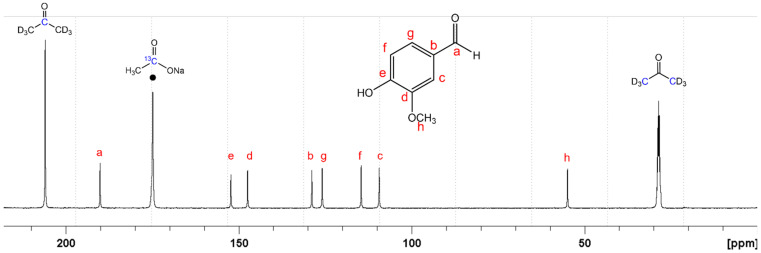
^13^C NMR (nuclear magnetic resonance) spectrum of a vanillin sample in acetone-d6, in the presence of CH_3_^13^CO_2_Na (●) as internal standard and Cr(acac)_3_ as relaxation agent. Peaks of solvent, standard, and analyte were assigned.

Spectroscopy analyses were performed following the experimental conditions previously set by Pironti et al. [19]. Vanillin, together with an internal standard and a relaxation reagent, was dissolved in the proper deuterated solvent. Sodium acetate was identified as an appropriate internal standard due to its relaxation time of 31.16 s and chemical shifts of 181.5 ppm, whereas chromium acetylacetonate was chosen as the relaxation reagent and acetone-d6 as solvent. The optimization of the experimental spectroscopic parameters and the use of an internal standard allowed us to reduce time analysis to 2 h. An example of a recorded spectrum is given in Figure 3.

For each vanillin sample, the carbon stable isotope ratio was obtained by the spectroscopic experiments as the mean value out of three different measurements. The first step was the integration of the spectra; the signal intensities of samples were normalized by the signal intensity of the carbonyl carbon of sodium acetate at 181.5 ppm. The equations used were:

(2) C13R=mass of internal standard (g/mol)molar mass of internal standard (g/mol)

(3) C13S=C13R×Is

(4) C13C=∑C13S

(5) C13C12=C13CCtot−C13C

^13^C_R_ is the molar carbon of the internal standard (Equation (2)), while ^13^C_S_ is the molar ^13^C of the sample, calculated by Equation (3) as the product of the internal standard and the molar carbon. Using this equation, the ^13^C_S_ of each vanillin signal was calculated, and the molar carbon sum ^13^C_C_ was obtained by adding all of the ^13^C_S_ values (Equation (4)). Finally, ^13^C/^12^C was calculated by Equation (5). 

The carbon isotope ratio ^13^C/^12^C values obtained from spectroscopic analysis of all samples of vanillin are reported in Table 1.

In line with the aim of this study, δ^13^C was calculated using the carbon stable isotope ratio obtained by spectroscopic analysis, and the reliability of this innovative methodology was assessed by comparing the obtained results with the δ^13^C measured by IRMS. The results of all the measurements (as the mean values of three determinations for each sample) with their standard deviation are reported in Table 1. Based on the δ^13^C range of values (from −38 to −25‰), a synthetic origin was established for the first eight considered samples. Moreover, the methodology is also applicable to the measurement of δ^13^C values of vanillin samples of natural origin, since for sample 9 (used as reference of a natural vanillin sample having a known δ^13^C value), a value close to the real one was recorded.

**Figure 4:**
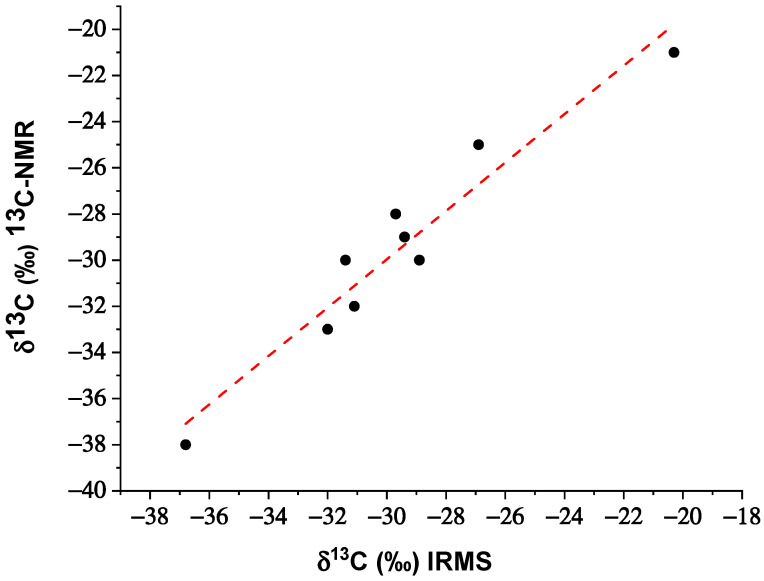
Correlation plot between δ^13^C values (‰) obtained with IRMS and ^13^C-NMR.

Even though the ^13^C-NMR method is affected by a higher deviation standard than IRMS (±1 compared to ±0.1), the δ^13^C values recorded with the two techniques are close to each other’s values for all vanillin samples analyzed. To better evaluate the correlation between the data obtained with the two analytical methodologies, a correlation plot was reported (Figure 4); the x-axis represents the δ^13^C values (‰) measured by IRMS, while the y-axis represents the values obtained by ^13^C-NMR analysis.

**Figure 5:**
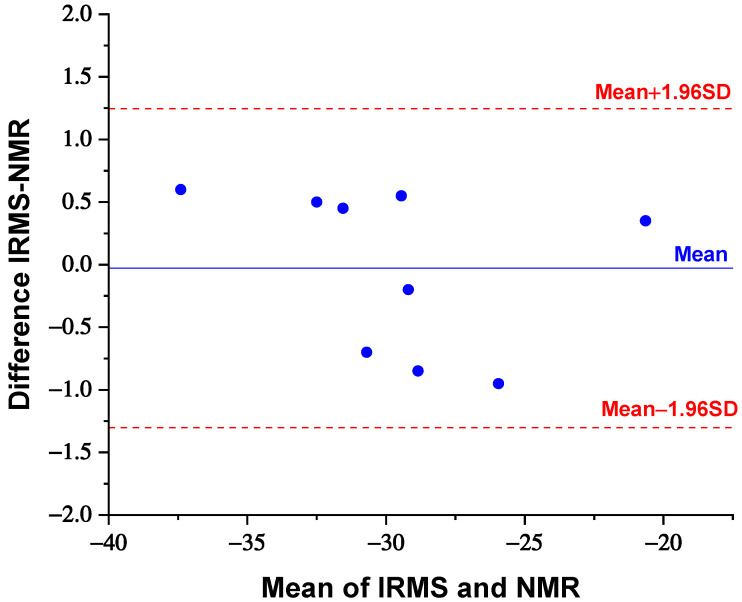
Altman–Bland plot of the difference in δ^13^C values between IRMS and NMR versus the mean of the data pairs (SD: standard deviation).

Linear regression analysis reveals a positive and strong correlation between the two techniques (Pearson’s r correlation coefficient of 0.964, R^2^ of 0.929, and *p*-value < 0.001). Since a strong correlation does not guarantee a good agreement between two methods, a Bland–Altman graph was constructed by plotting the difference between the results of the two methods (IRMS-NMR) against their mean (Figure 5) with the aim to evaluate the agreement between IRMS and ^13^C-NMR. The dotted red line in Figure 5 represents the limit of agreement (mean ± 1.96SD), defined as the limit where 95% of the differences would lie if the two methods were in agreement. Since the differences between IRMS and NMR for all the samples are distributed within the limits of agreement, IRMS and ^13^C-NMR methodologies are in agreement, so they can be used interchangeably.

Furthermore, the results of ANOVA analysis showed there are not significant differences (*p*-value of 0.942) between the δ^13^C values obtained with the two methods (IRMS and NMR), while there are significant differences (*p*-value ≤ 0.01) between δ^13^C values of vanillin samples of a different origin (natural and synthetic). The distribution of data is symmetric (skewness value of 0.4046), and the coefficients of variation are from 0.27 to 0.49% for IRMS and from 2.7 to 5.5% for NMR.

These results suggest that this ^13^C-NMR-based methodology could be used as an alternative to IRMS for the measurement of the carbon stable isotope ratio in vanillin samples.

## 4. Discussion

Food frauds are a worldwide problem commonly associated with foods, food ingredients, and some highly processed foods. Many researchers investigated new trends in analytical methodologies to reveal food fraud in order to improve the detection of illegal products. In the recent literature, Portaluri et al. [20] introduced a new approach to investigate isotope vanillin intramolecular composition with site-specific NMR spectroscopy because of the growing interest in the market. In fact, this literature study reported the enhancement of SNIF-NMR using INEPT sequences modified as adiabatic to achieve a good result in terms of precision and reproducibility, even if the carbon stable isotope ratio was always related to the isotopic value of the matrix calculated by IRMS. In our study we investigated the possibility of using ^13^C-NMR spectroscopy in food fraud investigation such as vanilla compounds without the use of isotope mass spectrometry to avoid the influence of bulk material. In fact, in our previous study, we obtained interesting results in the determination of the carbon isotope ratio of inorganic carbonates and a small molecule, oxalic acid, with high accuracy (1.0‰). 

In the literature, investigations of vanillin sources were usually focused on a combination of two techniques, namely, IRMS and NMR spectroscopy, to obtain site-specific enrichment of ^13^C by merging the δ^13^C values measured by IRMS with the molar fraction of ^13^C in the spectra. In particular, IRMS provides a global isotope composition without distinction of the matrix and possible mixtures, while quantitative NMR measures isotope ratios at every single site. Although by separating and quantitating each isotopomer, IRM-NMR (isotope ratio measurement by NMR) resolves isotope fractionation at natural abundance for every position in target molecules, the isotopic value is affected by possible errors created during IRMS analysis. These technical limitations can sometimes make the detection of sophisticated frauds difficult. This is one of the disadvantages of IRMS techniques. 

On the other hand, the accurate determination of the δ^13^C values by NMR results is attractive when the use of an internal standard, such as sodium acetate, is involved. The ^13^C NMR spectrum of a vanillin sample is reported in Figure 2 to clarify the protocol of the method used. Vanillin presents eight well-separated signals, identified and attributed to the corresponding carbon sites of the molecule isotopomers in acetone-d6. All samples analyzed were classified as synthetic vanillin, because δ^13^C values ranged from −38 to −25‰, according to values reported by Akoka et al. [33]. The spectra recorded showed no impurities or by-products added in the samples investigated. The additional possibility of evaluating the presence of impurities is one of the most important advantages of spectroscopic analysis over IRMS, as the latter allows to analyze bulk material without discrimination. The stable carbon isotope ratio of sample 5 (δ^13^C of −38‰) was about 13–15‰ lower compared to the other samples; this value can be influenced by the isotopic composition of raw material and possible fraction processes during synthesis. It must be taken into account that some factors (e.g., the amount of each raw material, conditions, and timing of processes) will affect the conversion of the precursors into vanillin. Recent studies reported the classification of vanillin from synthetic sources (petrochemicals range from −36.2 to −24.9‰ and lignin from −28.7 to −26.5‰) and from biotechnologically derived sources, such as fungi, yeast, and/or bacteria. In our case, we can suppose that sample 5 is derived from rice, whose δ^13^C ranges from −37.9 to −35.4‰ [21]. 

The possibility of discriminating between natural, bionatural, and synthetic sources is strictly related to one of the most important characteristics of a methodology, such as the error value associated with the measurement. In this study, values were averaged over the three spectra, and the standard deviation was calculated as a precision indicator for measurement repeatability, set at 1‰. The proposed method has the advantage of being simple and accurate due to the use of an internal standard that allows one to determine the δ^13^C value of the sample by performing only the ^13^C-NMR measurement. To evaluate its feasibility, results of ^13^C-NMR spectroscopy were compared to those obtained by IRMS, because IRMS is a reference technique and accepted by the scientific community for this type of measurement. Notwithstanding that the described innovative analytical methodology for δ^13^C determination has a higher standard deviation than isotope ratio mass spectrometry (*σ* = 0.1‰), it could be useful in the discrimination between various sources of vanillin, considering that the ranges of ^13^C values for a single class are wide. In fact, as highlighted in Table 1, δ^13^C values of vanillin samples obtained by ^13^C-NMR spectroscopy were consistent with that measured via IRMS analysis, showing good agreement between techniques (as confirmed by the Altman–Bland plot in Figure 5). In addition, the results of statistical analysis (ANOVA) showed that there are no significant differences (*p*-value of 0.942) between the δ^13^C values measured with ^13^C-NMR spectroscopy and those recorded with IRMS.

The obtained results give us confidence that the proposed ^13^C-NMR method could be an alternative to common techniques for the analysis of carbon isotopic composition in vanillin samples at natural abundance.

## 5. Conclusions

In this work, the applicability and the enhancement of quantitative ^13^C-NMR spectroscopy for the determination of carbon isotopic composition, at natural abundance, of vanillin samples were evaluated. This analytical protocol is unprecedented to the best of our knowledge. Starting from the results of our previous work on small molecules, such as carbonates and oxalic acid, nine commercially available vanillin samples were analyzed using our recent application of ^13^C-NMR spectroscopy. The δ^13^C values obtained with this methodology were between −38 and −25‰, suggesting a synthetic origin for all the samples with unknown origin. The proposed method is simple and accurate (standard deviation of 1.0‰), and, due to the employment of an internal standard, it allows us to determine the δ^13^C value of vanillin samples by carrying out only the ^13^C-NMR measurement without the use of IRMS. Statistical analysis proved that the results recorded with ^13^C-NMR spectroscopy are in agreement with those obtained with IRMS, which is the reference technique for δ^13^C measurements. In conclusion, this study proved that ^13^C-NMR methodology can be used for the determination of the δ^13^C of vanilla matrices, offering a wider choice of techniques suitable for the analysis of carbon isotopic composition.

## Figures and Tables

**Table 1:** Carbon isotope ratio ^13^C/^12^C obtained by ^13^C-NMR isotopic analysis and comparison of carbon isotopic values of vanillin samples obtained by ^13^C-NMR and IRMS analyses.

Sample	^13^Cs/^12^Cs (NMR)	δ^13^C (‰) ^13^C-NMR	σ	δ^13^C (‰) IRMS	σ	Classification
1	0.0115968	−32	±1	−31.1	±0.1	synthetic
2	0.0115631	−29	±1	−29.4	±0.1	synthetic
3	0.0115743	−30	±1	−31.4	±0.1	synthetic
4	0.0115743	−30	±1	−28.9	±0.1	synthetic
5	0.0116642	−38	±1	−36.8	±0.1	synthetic
6	0.0115518	−28	±1	−29.7	±0.1	synthetic
7	0.0115181	−25	±1	−26.9	±0.1	synthetic
8	0.0116080	−33	±1	−32.0	±0.1	synthetic
9 *	0.0110027	−21	±1	−20.3	±0.1	natural

* Sample of natural vanillin (known δ^13^C value) used for assessing the reliability of the method. NMR: nuclear magnetic resonance; IRMS: isotope ratio mass spectrometry.

## Data Availability

Not applicable.
